# Colorectal Cancer Risk Following Cholecystectomy: An Updated Systematic Review

**DOI:** 10.3390/cancers17193114

**Published:** 2025-09-24

**Authors:** Pierre-Henri Nelis, Stefano Grotto, Kenza Azra Ibis, Nashaira Nahar, Azzadinne Belhaj, Myriam Benhadda, Aude Vanlander, Nouredin Messaoudi

**Affiliations:** Department of Hepatopancreatobiliary Surgery, Vrije Universiteit Brussel, Universitair Ziekenhuis Brussel and Europe Hospitals, 1090 Brussels, Belgium; pierre-henri.paul.t.nelis@vub.be (P.-H.N.); stefano.grotto@uzbrussel.be (S.G.); kenza.azra.ibis@vub.be (K.A.I.); nashaira.nahar@uzbrussel.be (N.N.); azzadinne.belhaj@uzbrussel.be (A.B.); md.bmyriam@gmail.com (M.B.); aude.vanlander@uzbrussel.be (A.V.)

**Keywords:** cholecystectomy, colorectal, cancer, cohort study

## Abstract

**Background/Objectives:** Cholecystectomy (CE) is among the most commonly performed surgical procedures worldwide. While it effectively treats gallstone disease, concerns have been raised about a potential long-term association with colorectal cancer (CRC), given overlapping risk factors and post-surgical physiological changes. Previous studies have reported inconsistent findings. This updated systematic review aimed to reassess the association between CE and CRC risk by incorporating the most recent evidence. **Methods:** In accordance with PRISMA 2020 guidelines, a systematic literature search was conducted in PubMed, Embase, Medline, Web of Science, and the Cochrane Library for studies published after May 2022. Eligible studies were observational cohort studies reporting relative risk estimates for CRC following CE. Data were extracted manually, and study quality was assessed using the NewcastleOttawa Scale (NOS). Only high-quality studies were included to update the systematic review. Publication bias was assessed using funnel plots and Egger’s test. **Results:** Out of 156 identified records, three new high-quality cohort studies met the inclusion criteria and were added to the 18 studies from the previous review, resulting in a total of 21 studies. The findings were heterogeneous: while no consistent association with overall CRC risk was observed, several studies reported an elevated risk of proximal (right-sided) colon cancer following CE. Egger’s test indicated no significant publication bias (*p* = 0.50). **Conclusions:** This updated systematic review suggests a potential association between CE and an increased risk of proximal colon cancer; however, the evidence remains inconclusive. Further prospective studies with robust confounder control and detailed tumor location-specific analyses are warranted to clarify causality and guide future screening strategies.

## Simple Summary

Cholecystectomy, or gallbladder removal surgery, is commonly performed to treat gallstones. While concerns have been raised about its potential link to colon cancer, past studies offer mixed findings. This updated systematic review incorporates recent observational cohort studies to further examine the association. Overall, no significant increase in colon cancer risk was observed; however, some evidence suggests a possible elevated risk for right-sided colon cancer. To better understand this potential link, large population-based studies using national databases are needed. These could help guide follow-up care and inform screening strategies for patients after gallbladder removal.

## 1. Introduction

Cholecystectomy (CE) is among the most frequently performed abdominal operations worldwide, particularly in regions with a high prevalence of gallstone disease. It is the standard treatment for symptomatic cholelithiasis, recurrent biliary colic, and acute cholecystitis [1]. While its short-term safety and efficacy are well established, the potential long-term health consequences of CE remain a subject of debate. In particular, concerns have been raised regarding a possible association with colorectal cancer (CRC).

CRC represents a major global health burden. It is the third most commonly diagnosed cancer and the second leading cause of cancer-related mortality, responsible for nearly 900,000 deaths annually. Incidence rates continue to rise, largely attributable to modifiable factors associated with a Westernized lifestyle, including obesity, low dietary fiber intake, physical inactivity, and alcohol consumption [2]. Identifying additional medical or surgical exposures that may modify CRC risk is therefore of significant clinical importance, particularly given the growing population of patients who have undergone CE.

Several mechanisms provide biological plausibility for a CE–CRC association. Removal of the gallbladder alters the enterohepatic circulation of bile acids, leading to continuous exposure of the intestinal mucosa to secondary bile acids, such as deoxycholic acid and lithocholic acid, which possess carcinogenic properties [3,4]. These compounds can induce oxidative stress, DNA damage, and activation of oncogenic signaling pathways. In addition, CE has been shown to influence gut microbiota composition, resulting in dysbiosis that may promote a pro-carcinogenic colonic environment. These changes appear most pronounced in the proximal colon, which may explain reports of site-specific increases in CRC risk [5,6].

Epidemiological studies investigating this association have produced conflicting results. Several large cohort studies have found no significant increase in overall CRC risk following CE [7], while others have reported a modest elevation, often limited to right-sided colon cancers [8]. Variability in study design, population demographics, follow-up duration, and adjustment for confounding factors such as obesity and smoking likely contribute to these inconsistencies. Meta-analyses and systematic reviews have similarly yielded inconclusive findings, though some suggest a potential tumor site–specific effect [9].

Since the most recent systematic review by Lin Mu et al. in 2023, multiple large-scale observational cohort studies have been published, offering new insights [9]. Updating the evidence base is necessary to clarify whether CE represents an independent risk factor for CRC or whether observed associations reflect shared risk exposures.

The present systematic review therefore re-examines the association between CE and CRC by incorporating the most recent evidence into existing literature. Particular attention is given to tumor location, study quality, and adjustment for confounding, with the aim of providing an updated and comprehensive assessment of whether CE should be considered in CRC risk evaluation and surveillance strategies.

## 2. Materials and Methods

### 2.1. Eligibility Criteria, Information Sources, and Search Strategy

This systematic review was conducted and reported in accordance with the Preferred Reporting Items for Systematic Reviews and Meta-Analyses (PRISMA) 2020 guidelines [10]. A comprehensive literature search was performed across five databases: PubMed, Embase, Web of Science, Medline, and the Cochrane Library, restricted to studies published after May 2022, which marked the endpoint of the most recent systematic review by Lin Mu et al., 2023 [9]. The same search strategy was applied, using the following keywords and Boolean operators: (“cholecystectomy” OR “cholecystectomies”) AND (“colorectal” OR “gastrointestinal”) AND (“carcinoma” OR “cancer” OR “neoplasm” OR “adenocarcinoma”) AND “cohort study”.

Studies were eligible for inclusion if they met the following criteria: (I) observational cohort study design, (II) cholecystectomy (laparoscopic or open) as the exposure, (III) colorectal cancer reported as a primary or secondary outcome, (IV) reporting hazard ratios (HR), relative risks (RR), and/or incidence rate ratios (IRR) with corresponding 95% confidence intervals (CI), or providing sufficient data to calculate them and (V) publication in English. Studies were excluded if they met any of the following criteria: (I) ineligible study design, (II) did not report effect measures with 95% CI, (III) did not report colorectal cancer as an outcome.

**Figure 1:**
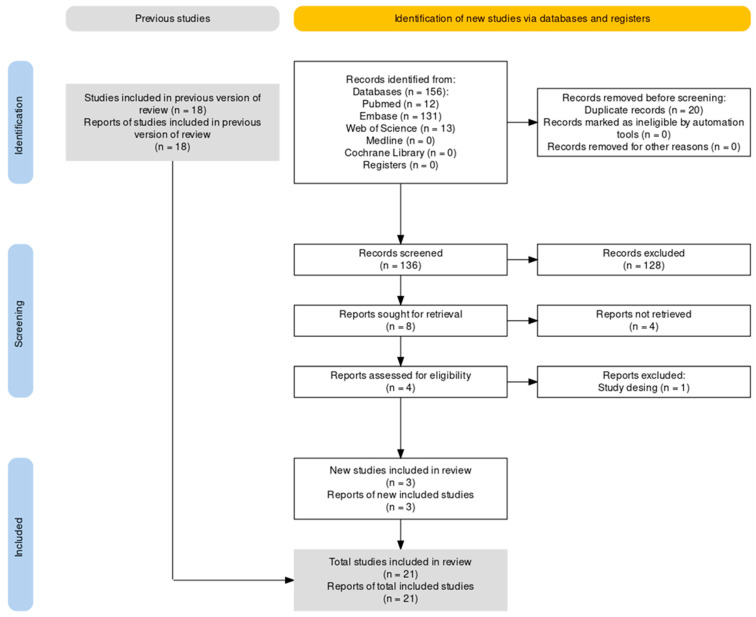
PRISMA flow diagram for updated systematic review [11].

Study selection was conducted manually without the aid of automation tools. Titles identified through the database searches were screened first, followed by abstracts to assess relevance. This process was performed manually, without the use of automation tools. Full-text articles of potentially eligible studies were then retrieved and assessed in detail against the inclusion and exclusion criteria. The complete study selection process is illustrated in the PRISMA flow diagram (Figure 1).

### 2.2. Data Collection and Synthesis

From each included study, the following data were extracted: first author, year of publication, country, study design, sample size, inclusion criteria, exposure (CE), comparator, outcome (CRC), effect estimates (RR, HR, IRR) with 95% CI, follow-up duration, any reported lag time and adjustments for confounders. Study quality was assessed using the Newcastle–Ottawa Scale (NOS). Studies were rated as high-quality (scores 7–9), moderate quality (scores 4–6), or low quality (scores 0–3). Only studies rated as high-quality were included to update the former systematic review [9].

Extracted data are presented in Table 1, organized chronologically to facilitate pattern recognition. When multiple effect estimates were provided, the most fully adjusted model was selected to minimize confounding. Heterogeneity among study findings was explored qualitatively by examining factors such as geographic location, study period, follow-up duration, and population characteristics (including subgroups, where applicable). Publication bias was assessed using a funnel plot and Egger’s regression test, both performed in Microsoft Excel (version Microsoft 365). A *p*-value of less than 0.05 was considered indicative of significant bias. No statistical sensitivity analyses were conducted.

To account for variability in confounder control, adjustments for key confounding factors associated with CRC risk were systematically extracted from each included study. As shown in Table 2, the adjustment status was assessed for age and sex, smoking, alcohol use, body mass index (BMI), dietary habits, diabetes mellitus (DM), inflammatory bowel disease (IBD), and other * comorbidity-related factors (physical activity, socioeconomic status (SES), hypertension (HT), dyslipidemia, hepatitis B and C infection, liver cirrhosis, stroke, coronary artery disease (CAD), chronic obstructive pulmonary disease (COPD) and chronic kidney disease (CKD). The frequency of confounder adjustment across 21 included studies is demonstrated in Table 3 to allow comparison on confounder adjustment across the included studies.

## 3. Results

### 3.1. Study Selection, Study Characteristics and Risk of Bias in Studies

The study selection process is outlined in the PRISMA flow diagram (Figure 1). A total of 156 records were identified through systematic searches of five electronic databases: PubMed, Embase, Web of Science, Medline, and the Cochrane Library. After the removal of 20 duplicates, 136 unique records were screened by title. Of these, 128 were excluded for not meeting the relevance criteria for this updated review.

Eight articles were selected for full-text retrieval and eligibility assessment. Of these, four were excluded: one was not published in English, three did not align with the study objective. Four full-text articles were assessed in detail, with one subsequently excluded due to an inappropriate study design. Ultimately, three newly identified studies met the inclusion criteria and were incorporated into this updated analysis, in addition to the 18 studies included in the prior systematic review by Lin Mu et al., 2023 [9], resulting in a total of 21 included studies.

Study characteristics are detailed in Table 1. The included studies were published between 1981 and 2025, and all investigated the association between cholecystectomy and the risk of colorectal cancer. The majority were large-scale retrospective cohort studies. Geographically, 11 studies originated from Europe, 9 from Asia, and 1 from the United States. Notably, all studies published since 2018 were conducted in Asia; the European studies were older.

### 3.2. Results of Synthesis

The findings from individual studies are summarized in Table 1, which reports the effect estimates along with corresponding 95% confidence intervals. A total of 21 cohort studies published between 1981 and 2025 were included, with sample sizes ranging from fewer than 2000 to more than 3 million participants and follow-up durations between 15 months and 33 years. Lag times varied from none to two years, with most studies applying at least a one-year exclusion. Study quality, assessed by the Newcastle–Ottawa Scale (NOS), ranged from 6 to 9, with the majority considered high quality.

Overall, 9 studies (43%) reported a statistically significant increase in CRC risk following cholecystectomy, 2 studies (10%) found a decreased risk, and 10 studies (48%) observed no significant association. Notably, most of the “no association” studies reported point estimates above 1.0, suggesting a trend toward increased risk, although their confidence intervals crossed unity and therefore did not reach statistical significance. Among the positive studies, several highlighted a stronger effect in the proximal colon. Conversely, those reporting decreased or null associations often applied longer follow-up durations or stricter lag periods, indicating that methodological differences may partly explain the heterogeneity. Potential sources of heterogeneity include: (I) geographic variation, (II) differences in adjustment for confounding factors such as diet, physical activity, and smoking, and (III) variations in follow-up duration and lag times, which may influence the detection and timing of CRC diagnosis.

To better understand the variability in the study results, the extent to which adjustments were made for known confounders for CRC risk was assessed. As shown in Table 2, all included studies adjusted for age and sex. However, adjustment for lifestyle and clinical confounders such as smoking, alcohol consumption, BMI, dietary habits, and comorbidities (e.g., diabetes, inflammatory bowel disease) was not consistent. Fourteen studies only adjusted for age and sex, while only a few studies took more than five confounding factors into account. This heterogeneity in the control of confounding factors likely contributes to the differences in the reported effect estimates and underscores the need for standardized adjustment in future research.

### 3.3. Reporting Biases and Certainty of Evidence

**Figure 2:**
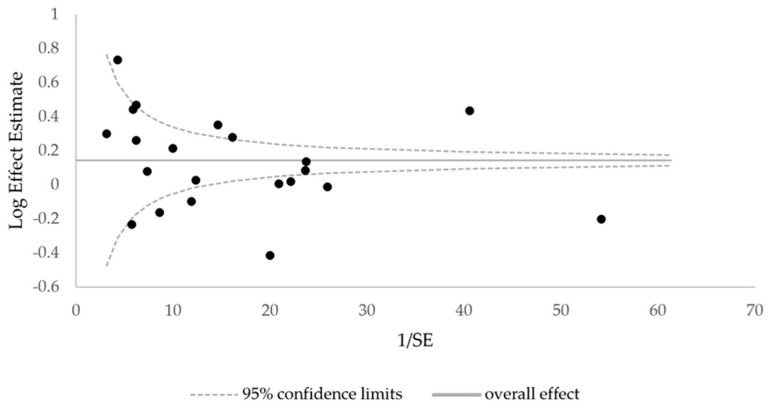
Funnel plot of the 21 included studies to assess publication bias. SE, standard error.

Assessment of publication bias using a funnel plot and Egger’s regression test (Figure 2) revealed no statistically significant evidence of bias (*p* = 0.502). However, the possibility of selective outcome reporting cannot be excluded, and studies reporting null or negative findings may be underrepresented in the published literature.

Overall, the certainty of the evidence regarding the association between cholecystectomy and colorectal cancer is considered moderate, though the association appears potentially more pronounced for proximal (right-sided) colon cancer.

## 4. Discussion

This updated systematic review evaluated the association between cholecystectomy (CE) and colorectal cancer (CRC) risk by integrating evidence from three newly identified cohort studies with the prior systematic review by Lin Mu et al. [9]. In total, 21 studies spanning more than four decades and multiple geographic regions were included. Despite this breadth, the findings remain heterogeneous, reflecting persistent uncertainty regarding the relationship between CE and CRC. While some studies reported a modest increase in overall CRC risk, particularly in the proximal colon, others found no significant association or even a decreased risk [9,17,19,23]. This heterogeneity underscores both the complexity of the CE–CRC relationship and the methodological limitations of the existing literature.

Post-CE changes in bile acid metabolism may contribute to the pathogenesis of CRC. Altered bile acid composition and increased exposure of the colonic mucosa to secondary bile acids such as deoxycholic acid (DCA) and lithocholic acid (LCA)—which exert carcinogenic effects through DNA damage, oxidative stress, immune modulation, microbiome changes, and Wnt/β-catenin pathway activation—offer biological plausibility for a CE-CRC link [3,4]. These effects appear most pronounced in the proximal colon, where bile acid concentration is higher and 7α-dehydroxylase activity, responsible for producing secondary bile acids, is more active [5,6].

A second pathophysiological mechanism involves post-CE changes in gut microbiota. The acceleration of enterohepatic circulation and altered pH may lead to dysbiosis, characterized by decreased beneficial bacteria such as Lactobacillus and Bifidobacterium and increased pathogenic species like Fusobacterium nucleatum, creating a pro-carcinogenic microenvironment [5,32,33]. These microbial and metabolic changes are more evident in long-term CE patients (>5 years) [34].

The anatomical location of CRC is increasingly recognized as clinically relevant. Several studies stratifying by tumor site reported a stronger association between CE and right-sided colon cancers. This is supported by differences in embryological origin, luminal content, and molecular mutations between right- and left-sided tumors [35,36,37,38]. Right-sided cancers more often harbor KRAS, PIK3CA, BRAF, and MET mutations, while left-sided ones show more TP53, SMAD4, and NRAS alterations [38]. However, some recent studies also reported a left-shift in tumor location after CE [13], warranting further investigation.

It is critical to recognize that CE and CRC share multiple risk factors, including obesity, low dietary fiber intake, sedentary lifestyle, and alcohol consumption [36,39,40,41]. Observed associations may therefore reflect residual confounding rather than a direct causal effect of CE. Nevertheless, some evidence suggests that CE itself could function as an independent risk factor, with a magnitude comparable to other modifiable lifestyle exposures [12,41].

A major source of heterogeneity across the included studies lies in the inconsistency of confounder adjustment. Although age and sex were universally accounted for, far fewer studies adjusted for lifestyle and metabolic factors such as BMI, smoking, alcohol use, or dietary habits. Table 2 and Table 3 highlight this variability. For example, Kim M et al. [12] incorporated smoking status into their models and demonstrated a strong interaction between CE and smoking, whereas Kim Y et al. [3] did not adjust for smoking. Such differences in analytic strategy likely explain part of the divergent results reported, even within the same geographical setting.

To enhance comparability, a standardized approach to confounder control is required. Based on current evidence, a minimum adjustment set should include age and sex as baseline epidemiological variables, BMI, smoking, and alcohol use as modifiable lifestyle factors, and diabetes mellitus and inflammatory bowel disease as comorbidities strongly associated with both gallstone disease/CE and CRC. These variables are consistently recognized as independent risk factors for both gallstone formation and colorectal carcinogenesis, making them critical elements in any analytic model.

The selection of this set is also supported by practical considerations. Age and sex are routinely available in clinical and administrative databases, while BMI, smoking, and alcohol use are relatively straightforward to collect and standardize across large populations. Diabetes mellitus and IBD are common comorbidities with well-established links to gastrointestinal pathology, and their inclusion would strengthen adjustment models considerably. Together, these factors represent a feasible yet robust minimum standard for confounder control in CE–CRC research.

Although dietary habits are known to play an important role in the development of both gallstone disease and CRC [35,42,43], their reliable assessment in large-scale epidemiological studies remains challenging. Self-reported dietary data are prone to recall bias and inconsistencies, and objective measures are resource-intensive, limiting their use in routine adjustment. For this reason, diet may be more suitable for dedicated prospective studies rather than as a core component of minimum confounder adjustment in population-based research. Adoption of a standardized adjustment set, as proposed here, would substantially reduce heterogeneity in reported outcomes and enhance the strength and interpretability of future evidence.

More specifically, patients with IBD may represent a subgroup at particular risk. IBD is a well-established risk factor for CRC, and patients with Crohn’s disease in particular have an elevated prevalence of gallstones [44,45]. In this context, CE may compound the pro-inflammatory environment and immune dysregulation already present, potentially increasing susceptibility to CRC. Although few studies have specifically investigated CE in the IBD population, this group warrants further attention in future research. Subgroup analyses in large national cohorts could clarify whether CE further modifies CRC risk in IBD patients, which may have implications for surveillance strategies.

The conflicting results reported earlier by Kim Y et al. [3] and Kim M et al. [12], two large population-based cohort studies from South Korea, highlight the complexity of the CE–CRC association, even within similar sociodemographic and genetic populations. Kim Y et al. observed a transient increase in CRC incidence within 1–5 years of CE, followed by a significantly reduced long-term risk in men after more than 10 years of follow-up. In contrast, Kim M identified CE as a major epidemiological risk factor for CRC, particularly among ex-smokers and current smokers.

Several methodological differences may explain these divergent findings. First, Kim M adjusted for smoking and demonstrated that CE may act synergistically with smoking to increase CRC risk, while Kim Y did not account for smoking. Second, Kim Y conducted a time-stratified analysis that revealed risk reduction after a decade, whereas Kim M focused more on short- to intermediate-term outcomes. Third, differences in adjustment for other potential confounders, such as alcohol us and diabetes, may also have contributed. Taken together, these findings suggest that CE may not uniformly increase CRC risk but could modify risk in combination with other exposures. This highlights the need for standardized confounder adjustment, subgroup analyses, and adequate follow-up in future research.

The timing of risk after CE appears to be an important determinant of the observed association with CRC. Several studies have reported a transient increase in incidence within the first 1–5 years after surgery, with subsequent normalization or even reduction in risk over longer follow-up [18,20]. This temporal pattern suggests that the physiological and microbiological changes induced by CE may exert early but not sustained carcinogenic effects.

However, the exact period of elevated risk varies considerably between studies. Kim Y et al. observed a higher incidence of CRC during the first one to three years post-CE, followed by a gradual decline to baseline levels after five to ten years [3]. Other analyses restricted the increased risk to the first year after surgery, after which the association disappeared [18,30]. These early elevations may reflect detection bias or the diagnosis of pre-existing or synchronous cancers that became apparent during the perioperative or immediate postoperative period, rather than a causal effect of CE itself.

In contrast, some studies reported a longer duration of increased risk, persisting for two, five, or even ten years after CE [20,21,22]. Johansen et al. noted a persistent excess risk extending through the entire 16-year follow-up period [25]. Conversely, other cohorts documented stabilization or even a decline in CRC risk over time. Adami et al. reported a decreased risk lasting up to 17 years of follow-up [29], while Chen et al. observed a protective effect as early as six months after surgery [17]. Late risk elevations have also been described: Ekbom et al. found no overall increase but reported a higher incidence in women starting 15 years post-CE [26], whereas Nielsen et al. observed an increased risk in men already within the first year [28].

This heterogeneity in temporal patterns highlights the critical importance of both lag time definitions and adequate follow-up duration. Applying an appropriate lag period is essential to exclude cancers that were likely pre-existing at the time of surgery. Indeed, when short-term diagnoses are excluded, the previously observed associations between CE and CRC often attenuate or disappear [3,17,18,22,30]. On the other hand, overly short follow-up may fail to capture long-term trends, while excessively long lag times risk overlooking genuine short-term effects. Standardization of lag time and follow-up reporting is therefore essential to distinguish between temporary, potentially biased risk elevations and true long-term associations.

While many studies have reported an increased incidence of CRC following CE, several others have observed no association or even a decreased risk [3,14,17,18,19,22,24,26,28,29,30,31]. Several explanations may account for these findings. First, patients undergoing CE often receive more frequent medical attention, which may facilitate the early detection and removal of adenomatous polyps, thereby reducing long-term CRC incidence [17]. Second, gallstone disease and chronic cholecystitis are associated with prolonged biliary inflammation and bile stasis, both of which may promote colorectal carcinogenesis; removal of the gallbladder could therefore eliminate a potential source of pro-carcinogenic exposure [9,35]. Third, the application of lag times in some studies has attenuated the initially observed risk, indicating that early cases may have represented pre-existing or synchronous cancers rather than a causal effect of CE. Finally, heterogeneity in confounder adjustment may also explain differences, as studies adjusting more rigorously for lifestyle and metabolic factors often reported no independent association between CE and CRC [3,17,18,22,30]. Together, these findings suggest that CE may not invariably increase CRC risk and that study design and follow-up are critical in interpreting outcomes.

Despite including high-quality studies based on the Newcastle–Ottawa Scale, several limitations should be noted. Most included studies were retrospective and observational in nature, limiting causal inference. Many older European studies had small cohorts, while more recent data come predominantly from Asia, potentially limiting generalizability. Although all studies adjusted for age and sex, subgroup analyses by tumor location, age, and sex were often not reported. In addition, lag times and follow-up durations varied considerably, affecting comparability. Few studies clearly reported the indication for CE, making it difficult to separate risks related to gallstones from those attributable to the surgery itself. Although a quantitative synthesis or subgroup meta-analysis would strengthen clinical applicability, substantial heterogeneity in study design, follow-up duration, lag periods, outcome definitions, and confounder adjustment limited the feasibility of a formal pooled analysis.

Future research should focus on large-scale, prospective cohort studies with rigorous control of confounders, standardized outcome definitions, and sufficient lag and follow-up periods. Recent European-based studies are particularly lacking, and additional data from these regions would improve generalizability. Subgroup analyses by tumor location, sex, age, and comorbidities such as IBD are essential to understanding heterogeneous risk patterns. Integration of biological markers, including bile acid profiles, microbiome composition, and molecular tumor characteristics, could further elucidate the mechanisms linking CE and CRC. Most importantly, we propose adoption of a minimum confounder adjustment set (age, sex, BMI, smoking, alcohol use, diabetes mellitus, and IBD) in all future studies. These variables are strongly and independently associated with both gallstone disease and CRC, are relatively easy to collect, and would substantially improve comparability across cohorts. Finally, longitudinal stratification by time since surgery should become routine in future CE–CRC research. Systematic reporting of risk estimates for intervals such as <1 year, 1–5 years, and >10 years after CE would allow for more accurate modeling of early, transient risk elevations versus long-term risk trajectories. Such standardized approaches are necessary to guide clinical practice and to determine whether CE should influence CRC surveillance strategies.

## 5. Conclusions

This updated systematic review suggests a potential association between CE and an increased risk of proximal CRC, although no consistent link with overall CRC risk was found. While biological mechanisms and regional trends support this association, the evidence remains inconclusive due to study heterogeneity and residual confounding. Recent large population-based studies even within the same country have reported conflicting results, underscoring the importance of standardized adjustment and long-term follow-up. Future high-quality prospective studies, particularly in underrepresented regions, are needed to clarify this relationship and guide potential surveillance strategies for at-risk populations.

## Figures and Tables

**Table 1:** Characteristics of the included studies assessing the association between cholecystectomy (CE) and colorectal cancer (CRC). The column ‘CRC risk’ indicates whether the observed risk was increased (↑), decreased (↓), or unchanged (–). ‘Interpretation’ provides a concise summary of study findings. Reported effect measures include Relative Risk, Hazard Ratio, and Incidence Rate Ratio, as provided by each study. Follow-up duration and lag time are reported consistently across studies. Study quality was assessed using the Newcastle–Ottawa Scale (NOS; maximum score 9). Abbreviations: CE, cholecystectomy; CRC, colorectal cancer; NOS, Newcastle–Ottawa Scale; RR, relative risk; HR, hazard ratio.

Author (Year, Ref., Country)	Design	Population (CE vs. Control)	Follow-Up (yrs)	Lag Time	CRC Cases (CE vs. Control)	Effect Estimate (95% CI)	CRC Risk	Interpretation	NOS
Kim Y (2025, [3], Korea)	Retrospective cohort	715,872 CE vs. 1,431,728 controls	5–13	1 yr	4374 vs. 9299	HR 0.82 (0.80–0.86)	↓	Reduced long-term risk	9
Kim M (2024 [12], Korea)	Retrospective cohort	174,874 CE vs. 174,874 controls	≤9	1 yr	1152 vs. 1018	HR 1.15 (1.06–1.25)	↑	Increased risk, esp. with smoking	9
Tsai (2023 [13], Taiwan)	Retrospective cohort	2404 CE vs. 112,948 controls	2–18.8	1 yr	221 vs. 3436	RR 1.42 (1.24–1.62)	↑	Elevated long-term risk	9
Choi (2022, [14], Korea)	Retrospective cohort	123,295 CE vs. 123,295 controls	4.6 (mean)	1 yr	1078 vs. 1003	RR 1.03 (0.88–1.21)	–	No association	8
Jung (2021, [15], Korea)	Retrospective cohort	408,769 CE patients	4.7 (mean)	1 yr	1773	RR 1.55 (1.48–1.63)	↑	Consistent increase	6
Kim (2020, [16], Korea)	Retrospective cohort	3588 CE patients	15 mo (0–146)	1 yr	21	RR 2.08 (1.28–3.17)	↑	Short-term increased risk	6
Chen (2020, [17], Taiwan)	Retrospective cohort	83,963 CE vs. 83,963 controls	Until CRC/death	6 mo	638 vs. 1170	RR 0.66 (0.60–0.73)	↓	Decreased risk	9
Lee (2018, [18], Korea)	Retrospective cohort	11,362 CE vs. 696,301 controls	13.7 (mean)	1 yr	34 vs. 4276	RR 0.79 (0.56–1.10)	–	No association	8
Shabanzadeh (2017, [19], Denmark)	Retrospective cohort	187 CE vs. 5327 controls	24.7 (mean)	None	11 vs. 183	RR 1.35 (0.73–2.51)	–	No association	8
Chen (2014, [20], Taiwan)	Retrospective cohort	5850 CE vs. 62,180 controls	Unclear	None	67 vs. 76	HR 1.56 (1.12–2.17)	↑	Elevated risk	7
Goldacre (2012, [21], UK)	Retrospective cohort	327,460 CE vs. 3,000,000 controls	Until CRC/death	2 yr	2245 vs. 3622	RR 1.24 (1.02–1.51)	↑	Modest increase	8
Goldacre (2005, [22], UK)	Retrospective cohort	39,254 CE vs. 334,813 controls	Until cancer/death	2 yr	505 vs. 3731	RR 1.02 (0.93–1.11)	–	No association	6
Shao (2005, 2005 [23], USA)	Retrospective cohort	55,960 CE vs. 574,668 controls	Until CRC/death	1 yr	297 vs. 2218	RR 1.32 (1.16–1.48)	↑	Elevated risk	7
Lagergren (2001, [24], Sweden)	Retrospective cohort	278,460 CE patients	12.1 (mean)	1 yr	3425	RR 1.01 (0.92–1.11)	–	No association	7
Johansen (1996, [25], Denmark)	Retrospective cohort	42,098 gallstone pts (72.4% CE)	1–16	1 yr	344 vs. 147	RR 1.09 (1.00–1.18)	↑	Slight increase	7
Ekbom (1993, [26], Sweden)	Retrospective cohort	62,615 CE patients	Until 1987	1 yr	633	RR 0.99 (0.92–1.07)	–	No association	6
Goldbohm (1993, [27], NL)	Prospective cohort	3500 (men/women with gallstones/CE)	3.3 (mean)	None	53 vs. 408	RR 1.60 (1.16–2.19)	↑	Elevated risk	8
Nielsen (1991, [28], Iceland)	Prospective cohort	3425 CE patients	8–33	None	57	RR 1.08 (0.82–1.40)	–	No association	7
Adami (1987, [29], Sweden)	Prospective cohort	16,439 CE patients	14–17	None	150	RR 0.91 (0.77–1.07)	–	No association	7
Adami (1983, [30], Sweden)	Prospective cohort	16,773 CE patients	11–14	None	130	RR 0.85 (0.68–1.07)	–	No association	6
Linos (1981, [31], Greece)	Retrospective cohort	1681 CE patients	13 (mean)	6 mo	42	RR 1.30 (0.95–1.78)	–	No association	7

**Table 2:** Summary of confounder adjustments in the included studies assessing the association between cholecystectomy and colorectal cancer (CRC). Shaded cells indicate that the variable was adjusted for; blank cells indicate no adjustment. The assessed confounders include age and sex, smoking, alcohol use, body mass index (BMI), dietary habits, diabetes mellitus (DM), and inflammatory bowel disease (IBD), and other * comorbidity-related factors (physical activity, socioeconomic status (SES), hypertension (HT), dyslipidemia, hepatitis B and C infection, liver cirrhosis, stroke, coronary artery disease (CAD), chronic obstructive pulmonary disease (COPD) and chronic kidney disease (CKD).

Study (Year)	Age and Sex	Smoking	Alcohol	BMI	Diet	DM	IBD	Other Comorbidities *
Kim Y et al. 2025 [3]								
Kim M et al. 2024 [12]								
Tsai et al. 2023 [13]								
Choi et al. 2022 [14]								
Jung et al. 2021 [15]								
Kim et al. 2020 [16]								
Chen et al. 2020 [17]								
Lee et al. 2018 [18]								
Shabanzadeh 2017 [19]								
Chen et al. 2014 [20]								
Goldacre 2012 [21]								
Goldacre 2005 [22]								
Shao 2005 [23]								
Lagergren 2001 [24]								
Johansen 1996 [25]								
Ekbom 1993 [26]								
Goldbohm 1993 [27]								
Nielsen 1991 [28]								
Adami 1987 [29]								
Adami 1983 [30]								
Linos 1981 [31]								

**Table 3:** Frequency of confounder adjustment across the 21 included studies. Most studies adjusted only for age and sex, while lifestyle and metabolic factors were rarely included. * “Other comorbidities” includes physical activity, socioeconomic status (SES), hypertension (HT), dyslipidemia, hepatitis B and C infection, liver cirrhosis, stroke, coronary artery disease (CAD), chronic obstructive pulmonary disease (COPD) and chronic kidney disease (CKD).

Confounder	No. of Studies Adjusted (Out of 21)
Age and Sex	21
Smoking	4
Alcohol use	4
BMI	3
Dietary habits	2
Diabetes mellitus	5
IBD	2
Other comorbidities *	6

## Data Availability

No new data were created. Data sharing is not applicable to this article.

## Abbreviations

The following abbreviations are used in this manuscript:

CE	Cholecystectomy
CRC	Colorectal cancer
NOS	Newcastle–Ottawa Scale
PRISMA	Preferred Reporting Items for Systematic Reviews and Meta-Analyses
CI	Confidence interval
SE	Standard error
RR	Relative risk
HR	Hazard ratio
IRR	Incidence rate ratio
BMI	Body mass index
SES	Socioeconomic status
HT	Hypertension
DM	Diabetes mellitus
CAD	Coronary artery disease
COPD	Chronic obstructive pulmonary disease
CKD	Chronic kidney disease
IBD	Inflammatory bowel disease
DCA	Deoxycholic acid
LCA	Lithocholic acid
